# Coverage of community-wide mass drug administration platforms for soil-transmitted helminths in Benin, India, and Malawi: findings from the DeWorm3 project

**DOI:** 10.1186/s40249-024-01241-0

**Published:** 2024-10-08

**Authors:** Arianna Rubin Means, Kristjana Hrönn Ásbjörnsdóttir, Katherine C. Sharrock, Sean R. Galagan, Kumudha Aruldas, Euripide Avokpaho, Félicien Chabi, Katherine E. Halliday, Parfait Houngbegnon, Gideon John Israel, Saravanakumar Puthupalayam Kaliappan, David Kennedy, Hugo Legge, William E. Oswald, Gokila Palanisamy, Elliott Rogers, Joseph Timothy, Emily Pearman, Rohan Michael Ramesh, James Simwanza, Jasmine Farzana Sheik-Abdullah, Mariyam Sheikh, Comlanvi Innocent Togbevi, Stefan Witek-McManus, Rachel L. Pullan, Robin Bailey, Khumbo Kalua, Moudachirou Ibikounlé, Adrian J. F. Luty, Sitara S. R. Ajjampur, Judd L. Walson

**Affiliations:** 1 Department of Global Health, University of Washington, Seattle, Washington USA; 2 Centre of Public Health Sciences, University of Iceland, Reykjavík, Iceland; 3 The DeWorm3 Project, University of Washington, Seattle, Washington USA; 4 The Wellcome Trust Research Laboratory, Division of Gastrointestinal Sciences, Christian Medical College, Vellore, India; 5 Institut de Recherche Clinique du Bénin, Abomey-Calavi, Bénin; 6 Department of Disease Control, Faculty of Infectious and Tropical Diseases, London School of Hygiene & Tropical Medicine, London, UK; 7 Global Health Division, International Development Group, Research Triangle Institute (RTI) International, Research Triangle Park, NC USA; 8 Division of Medicine, University College London, London, UK; 9 Department of Population Health, Faculty of Epidemiology and Population Health, London School of Hygiene & Tropical Medicine, London, UK; 10 Clinical Research Department, Faculty of Infectious and Tropical Diseases, London School of Hygiene & Tropical Medicine, London, UK; 11 Blantyre Institute for Community Outreach, Lions Sight First Eye Hospital, Blantyre, Malawi; 12 School of Population and Public Health, University of British Columbia, Vancouver, British Columbia Canada; 13 Tropical Infectious Diseases Research Center (TIDRC)Université d’Abomey-Calavi, Abomey-Calavi, Bénin; 14 Université Paris Cité, Institut de Recherche pour le Développement, MERIT, Paris, F-75006 France; 15 Department of International Health, Bloomberg School of Public Health, Johns Hopkins University, Baltimore, MD USA

**Keywords:** Soil-transmitted helminths, Mass drug administration, Albendazole, Community, Coverage

## Abstract

**Background:**

Soil-transmitted helminths (STH) affect approximately 1.5 billion people globally. The current STH control strategy is annual or twice-annual preventive chemotherapy, typically school-based deworming targeting children and women of reproductive age. Mathematical modeling suggests that it may be possible to interrupt STH transmission through high-coverage community-wide mass drug administration (cMDA). DeWorm3 is a cluster randomized trial testing cMDA for prevalence reduction and transmission interruption. The purpose of this study is to describe coverage of cMDA in study clusters over time and correlates of coverage at individual and cluster levels.

**Methods:**

From 2018–2020, DeWorm3 delivered six rounds of cMDA with 400 mg albendazole at sites in Benin, India, and Malawi. We report coverage, treatment uptake, and directly observed therapy across all rounds. Factors associated with coverage at the cluster level were identified using binomial generalized estimating equations, while factors associated with non-treatment at the individual level were identified using binomial mixed-effects models.

**Results:**

Coverage was high across all clusters and rounds, exceeding the WHO target of 75% in all sites and across all rounds (78% to 95%); cluster-level coverage tended to increase over time. Younger, unmarried, and migratory adults were more likely to be untreated at all sites; adult males were more likely to be untreated in Benin and Malawi. Among children, girls were more likely to be untreated, as were non-school-attending and migratory children. Higher adult education was associated with greater odds of non-treatment among adults, but lower odds among children in the household. Belonging to a less wealthy or minority language-speaking household was associated with non-treatment among both adults and children.

**Conclusions:**

It is possible to deliver community-wide MDA with high coverage. Unique individual and community-level factors influence treatment across settings, and these may be addressed through targeted programming.

*Trial Registration*: Field Studies on the Feasibility of Interrupting the Transmission of Soil-transmitted Helminths (STH), NCT03014167.

**Supplementary Information:**

The online version contains supplementary material available at 10.1186/s40249-024-01241-0.

## Background

Soil-transmitted helminths (STH) are neglected tropical diseases (NTDs) that infect approximately 1.5 billion people globally [1]. Infections with STH are associated with adverse outcomes including diarrhea, general malaise and weakness, while moderate to heavy infections are associated with malnutrition and chronic anemia [2]. Some publications have linked STH infection to reduced cognitive development and economic productivity, but the evidence for causality is mixed. [3] The current standard of care for STH focuses on the control of morbidity using annual or bi-annual preventive chemotherapy with anthelmintics to reduce infection intensity in children and women of reproductive age [4, 5]. Anthelmintics are typically delivered to pre-school-age and school-age children (PSAC and SAC) living in STH endemic areas, often via school-based delivery platforms that engage both teachers and formal and informal drug distributors as the primary implementers [4, 5]. However, in 2020, the World Health Organization (WHO) reported that only 42% of children at risk of STH globally were successfully treated with anthelmintics [1]. Evidence suggests that it may be possible to substantially reduce the prevalence of STH, and potentially to interrupt transmission, via community-wide mass drug administration (cMDA), whereby all age groups are treated [6–8].

If transmission interruption is achievable using cMDA, such an approach would reduce the presence of adult reservoirs of infection in the community and the risk of re-infection post-deworming and may ultimately allow for the cessation of STH treatment programs in some settings [9, 10]. In order for cMDA to interrupt transmission, it is likely necessary to achieve high MDA treatment coverage, encompassing both wide reach of cMDA and high treatment uptake among those reached [11–15]. Achieving and reliably estimating high cMDA coverage requires detailed understanding of the population living in at-risk areas, the population eligible for treatment, and multi-level factors associated with coverage that can be addressed to improve coverage during future rounds of cMDA. Evidence from NTD platforms indicates that cMDA coverage is influenced by individual-level social factors such as religious beliefs, gender norms, education, and household social dynamics [16, 17]. Coverage is also influenced by community-level factors including geographic location [18], community engagement and sensitization methods [19], and other attributes of cMDA delivery. However, accurately estimating coverage and understanding drivers of coverage is often limited by poor census data, incomplete reporting, data aggregation errors, and challenges in tracking individual participation longitudinally [20, 21].

DeWorm3 is a multi-site cluster-randomized trial testing the feasibility of interrupting transmission of STH via cMDA [22, 23]. Here we report cMDA treatment coverage observed over three years of implementation. We also investigate individual- and community-level correlates of coverage to identify opportunities to maintain or improve treatment coverage in future elimination campaigns.

## Methods

DeWorm3 was implemented in Benin, India, and Malawi. In Benin, the study site includes Comé town and the surrounding rural area of the Commune of Comé. The study area in India comprises two geographically distinct sub-sites within the state of Tamil Nadu, a plains area in Timiri and a tribal region in Jawadhu Hills. The Malawi site is on the Namwera plateau, a rural area within Mangochi district.

Each site includes 80,000 or more individuals, divided into 40 contiguous clusters each comprising at least 1650 individuals. Clusters were randomized 1:1 to intervention and control arms using restricted randomization to balance arms by baseline population and factors hypothesized to be strongly associated with STH transmission (e.g., age distribution). The primary outcome of the trial and additional details about cluster demarcation and trial design are detailed elsewhere [22].

From 2018 to 2020, intervention clusters received twice annual cMDA with a single dose of albendazole (six rounds total), delivered to individuals eligible under national guidelines (described below). Control clusters received standard-of-care school-based deworming (SBD), comprising bi-annual targeted treatment of PSAC and SAC (India) or annual targeted treatment of SAC (Benin and Malawi). More information about the study can be found in Supplementary Materials 1. To encourage high treatment uptake, community sensitization activities took place prior to MDA in close consultation and collaboration with National NTD Programs. Sensitization activities (Supplementary Materials 2) by cluster were documented in real time during each round of cMDA [24].

### Annual census

Annual censuses, conducted by study data collectors, enumerated the study population and established a denominator for cluster-level coverage estimates. Data were collected via Android devices running SurveyCTO software (Dobility, Inc.; Cambridge, MA, USA and Ahmedabad, India) and harmonized within a central database.

The census at study baseline (pre-intervention) documented and/or observed key sociodemographic and STH risk factors, collected information about individual household members, and recorded GPS coordinates of all structures in study sites. Household members were classified as migratory if they spent the majority of nights elsewhere in the past six months. Annual census updates verified births, deaths, and migration status of all individuals. Up to three attempts were made to reach households during each census. If new households were identified during cMDA or other study activities, they were included in sampling lists for future census updates.

Written informed consent (or oral consent with documented thumbprint in the presence of a witness) was provided by heads of household or other adult members of the household prior to each census.

### MDA delivery

In intervention clusters, twice-annual cMDA, delivered house-to-house by NTD program drug distributors accompanied by study data collectors, used treatment lists generated from the most recent census. Children were eligible for SBD at rounds 2, 4, and 6 (Benin and Malawi) or at all rounds (India). Those who were not treated via SBD could be treated via subsequent cMDA, scheduled to occur within two weeks of SBD.

Individuals were ineligible for treatment if they were < 12 months of age in Benin and India or < 24 months in Malawi, treated within the past two weeks (e.g., during SBD), pregnant in their first trimester, seriously ill, or had a history of adverse reactions to benzimidazoles. Drug distributors requested to directly observe treatment whenever household members were present. If a household member was absent, the drug distributor returned two more times. Thereafter, treatment for the individual was left with the head of household. Data collectors recorded reasons for non-treatment (e.g., not present, refused, etc.).

Treatment was documented using SurveyCTO. Digital dashboards displaying treatment coverage were reviewed by teams daily to guide mop-up activities and allocate resources [25].

### Statistical analysis

Data were analyzed using R (R Computing, Vienna, Austria) and Stata 17 (StataCorp, College Station, Texas).

### Treatment coverage

Treatment coverage in intervention clusters was assessed directly using MDA treatment registers. The primary definition of cMDA coverage, referred to as per-protocol coverage, comprised the proportion of censused and eligible individuals who received a dose of albendazole at each treatment round, whether via cMDA or SBD (Supplementary Materials 3). In March 2020, cMDA was cut short in four clusters in India due to COVID-19 lockdown orders, and these cluster rounds are excluded from analyses.

### Correlates of MDA coverage

Using MDA treatment register data, factors potentially associated with cluster-level per-protocol coverage and with individual-level treatment history across all six rounds of MDA were assessed in intervention clusters, stratified by site. Cluster-level models used generalized estimating equations with binomial distribution, autoregressive correlation structure, and robust standard errors, with a significance level of 0.05. Cluster-level factors including population density, number of drug distributors trained, and sensitization activities were assessed, and aggregate measures of clusters’ population demographics (e.g., proportion adult, migratory, speaking a minority language) were divided into two-to-five categories for inclusion in models based on site distributions. Base models assessed each factor adjusted only for MDA round, and the final fully adjusted model was selected using the quasi-likelihood under the independence model criterion (QIC), using the qic package in Stata [26]. Differences in proportion treated per one unit increase (dy/dx) were calculated for each factor included in the model.

Individual-level models assessed factors associated with non-treatment among censused, eligible individuals using a binomial mixed effects model with random intercepts for cluster and individual and random slopes to account for individual trends over six rounds of MDA, with a significance level of 0.05. Individual-level models were stratified into adults and children (including school-age young adults in India and Malawi). Both adult and child models included age, gender, migratory status, language and religion (minority vs. majority), household wealth quintile calculated using the Demographic Health Survey wealth index approach, and population density within a 0.5 km radius of the household. Adult models additionally included marital status and education level, while child models included school attendance and highest education achieved by an adult member of their household. Unadjusted models assessed each variable individually, while the fully adjusted model included mutual adjustment for all a priori specified variables.

## Results

### Study population

The study population comprised 20 intervention clusters per site (*n* = 60 clusters), ranging from 48,241 individuals in Benin to 68,457 in India at baseline (Table 1). Age distribution differed substantially between sites, with 77.4% of study area residents in India being adults, compared to 51.3% in Malawi. The population at the Benin site was the most diverse in terms of language and religion, with 10.6% speaking a minority language and 41.4% practicing a minority religion. Benin had the lowest proportion of individuals identified as migratory at baseline (1.5%, cluster range 0.4–4%) compared to 2.9% in India, and 3.8% in Malawi.

**Table 1:** Demographic characteristics of DeWorm3 intervention clusters from the baseline census

	Benin	India	Malawi
**Individual-level demographic data**	(*n* = 48,241)	(*n* = 68,457)	(*n* = 61,007)
Age: mean (SD)	23.2 (18.7)	32.9 (20.3)	21.6 (19.5)
Age category, *n* (%)			
Infants (< 1 year)	1319 (2.7)	850 (1.2)	2168 (3.6)
Pre-school-age children (1–4 years)	5775 (12.0)	4029 (5.9)	8687 (14.2)
School-age children (5–14 years)	13,146 (27.3)	10,578 (15.5)	18,747 (30.7)
Adults (15 + years)	27,963 (58.0)	53,000 (77.4)	31,321 (51.3)
Unknown age	38 (< 0.1)	0 (0.0)	84 (< 0.1)
Sex, *n* (%)			
Male	23,188 (48.1)	34,153 (49.9)	28,892 (47.4)
Female	25,052 (51.9)	34,300 (50.1)	32,114 (52.6)
Other	1 (< 0.1)	4 (< 0.1)	1 (< 0.1)
School attendance, among children and young adults eligible for standard-of-care MDA, *n* (%)^a^			
Not currently attending school	7498 (37.0)	6770 (31.6)	13,282 (36.8)
Currently attending school	10,764 (53.2)	14,656 (68.4)	22,820 (63.2)
Unknown school attendance	1978 (9.8)	0 (0.0)	31 (< 0.1)
Education level, among adults not eligible for standard-of-care MDA, *n* (%)^b^			
No education/less than primary	8638 (30.9)	15,576 (33.1)	10,352 (41.8)
Any primary/any middle school	5041 (18.0)	14,215 (30.2)	11,283 (45.5)
Any secondary education/higher secondary education	8492 (30.4)	11,555 (24.6)	2149 (8.7)
Any higher education/tertiary education	2561 (9.2)	5437 (11.6)	42 (< 0.1)
Other education level	261 (0.9)	33 (< 0.1)	105 (< 0.1)
Unknown education level	2970 (10.6)	215 (< 0.1)	859 (3.5)
Marital status, among adults 15 + years of age, *n* (%)			
Unmarried	10,035 (35.9)	14,387 (27.1)	11,498 (36.7)
Married	14,454 (51.7)	35,165 (66.3)	17,115 (54.6)
Married (polygamous)	634 (2.3)	N/A	2191 (7.0)
Unknown marital status	2840 (10.2)	3448 (6.5)	517 (1.7)
Migratory status, *n* (%)			
Migratory^c^	715 (1.5)	1976 (2.9)	2342 (3.8)
Non-migratory	47,526 (98.5)	66,481 (97.1)	58,665 (96.2)
Wealth quintile, *n* (%)^d^			
Lowest quintile	8947 (18.5)	11,616 (17.0)	10,858 (17.8)
Low quintile	8354 (17.3)	12,902 (18.8)	11,595 (19.0)
Middle quintile	9541 (19.8)	13,898 (20.3)	12,583 (20.6)
High quintile	10,205 (21.2)	15,401 (22.5)	12,778 (20.9)
Highest quintile	11,194 (23.2)	14,640 (21.4)	13,193 (21.6)
Household religion, *n* (%)^e^			
Majority religion	28,252 (58.6)	65,826 (96.2)	57,687 (94.6)
Minority religion	19,965 (41.4)	2616 (3.8)	3,317 (5.4)
Unknown religion	24 (< 0.1)	15 (< 0.1)	3 (< 0.1)
Household language, *n* (%)^f^			
Majority language	43,104 (89.4)	66,263 (96.8)	58,633 (96.1)
Minority language	5122 (10.6)	2194 (3.2)	2371 (3.9)
Unknown language	15 (< 0.1)	0 (0.0)	3 (< 0.1)
Population density within 0.5 km of the household (km^2^), *n* (%)			
< 500	5465 (11.3)	14,115 (20.6)	8053 (13.2)
500–999	5157 (10.7)	19,221 (28.1)	9798 (16.1)
1 000–2 499	7574 (15.7)	25,804 (37.7)	32,156 (52.7)
2 500–4 999	11,714 (24.3)	7633 (11.2)	10,975 (18.0)
5 000 +	18,268 (37.9)	1627 (2.4)	0 (0.0)
Unknown population density	63 (< 0.1)	57 (< 0.1)	25 (< 0.1)
**Cluster-level demographic data**	(*n* = 20)	(*n* = 20)	(*n* = 20)
Proportion in cluster: median (range)			
Adult	59% (54–66%)	80% (70–82%)	55% (52–58%)
Male	48% (45–50%)	50% (49–52%)	47% (45–50%)
Speaking minority language^f^	8% (0.4–27%)	3% (0–9%)	2% (0.2–17%)
Practicing minority religion^e^	34% (12–81%)	2% (0–23%)	3% (0.3–27%)
Polygamous	2% (2–6%)	N/A	7% (4–11%)
Migratory^c^	1% (0.4–4%)	2% (0.6–7%)	3% (0.8–8%)
Mean population density within 0.5 km of the household/km^2^), median (range)	4149 (4880–8058)	1114 (323–4206)	1445 (587–3843)
Number drug distributors trained in cluster prior to MDA1, median (range)	4 (2–7)	8 (4–15)	1 (1–4)
**Sensitization activities: median (range) number of activities in clusters prior to MDA1**			
Community meetings	1 (1–1)	2 (1–3)	3 (1–7)
Public dialogue events	3 (3–12)	0	1 (1–1)
Printed IEC materials	26 (14–44)	67 (42–96)	11 (11–11)
Door to door visits	0	0	290 (92–574)
Radio	45 (45–45)	0	0
TV	0	0	0
Newspaper	0	0	0
Other mass media	0	0	0

*MDA* mass drug administration, *SD* standard deviation, *WASH* water, sanitation and hygiene, *N/A*: Not applicable

^a^ < 20 years of age in India and Malawi and < 15 years of age in Benin

^b^20 + years of age in India and Malawi and 15 + years of age in Benin

^c^Lived in the household less than six months in the previous year

^d^SES quintile calculated using Principal Components Analysis, taking the first principal component, on a list of household assets including house characteristics and appliance, technology, and livestock ownership (with additional fertilizer and water, sanitation and hygiene [WASH] access variables in Malawi only)

^e^Majority religion in the study area: Christianity in Benin; Islam in Malawi; Hinduism in India. Minority religions: Islam, Voodoo, or traditional religion in Benin; Christianity or other in Malawi; Christianity, Islam, or other in India

^f^Majority language in the study area: Pedah, Sahoue, Watchi, Mina, Adja, or Xwla in Benin; Chiyao in Malawi; Tamil in India. Minority language: Fon or other in Benin; Chichewa or other in Malawi; Telegu, Urdu, Hindi or other in India

Mean population density within 0.5 km of households was highest at the Benin site, which includes the town of Comé, at a cluster median of 4149 individuals/km^2^. However, median population density differed substantially within sites, with a greater than ten-fold difference across clusters in Benin (488–8058) and India (323–4206) and a six-fold difference in Malawi (587–3843).

### Treatment coverage

**Fig. 1:**
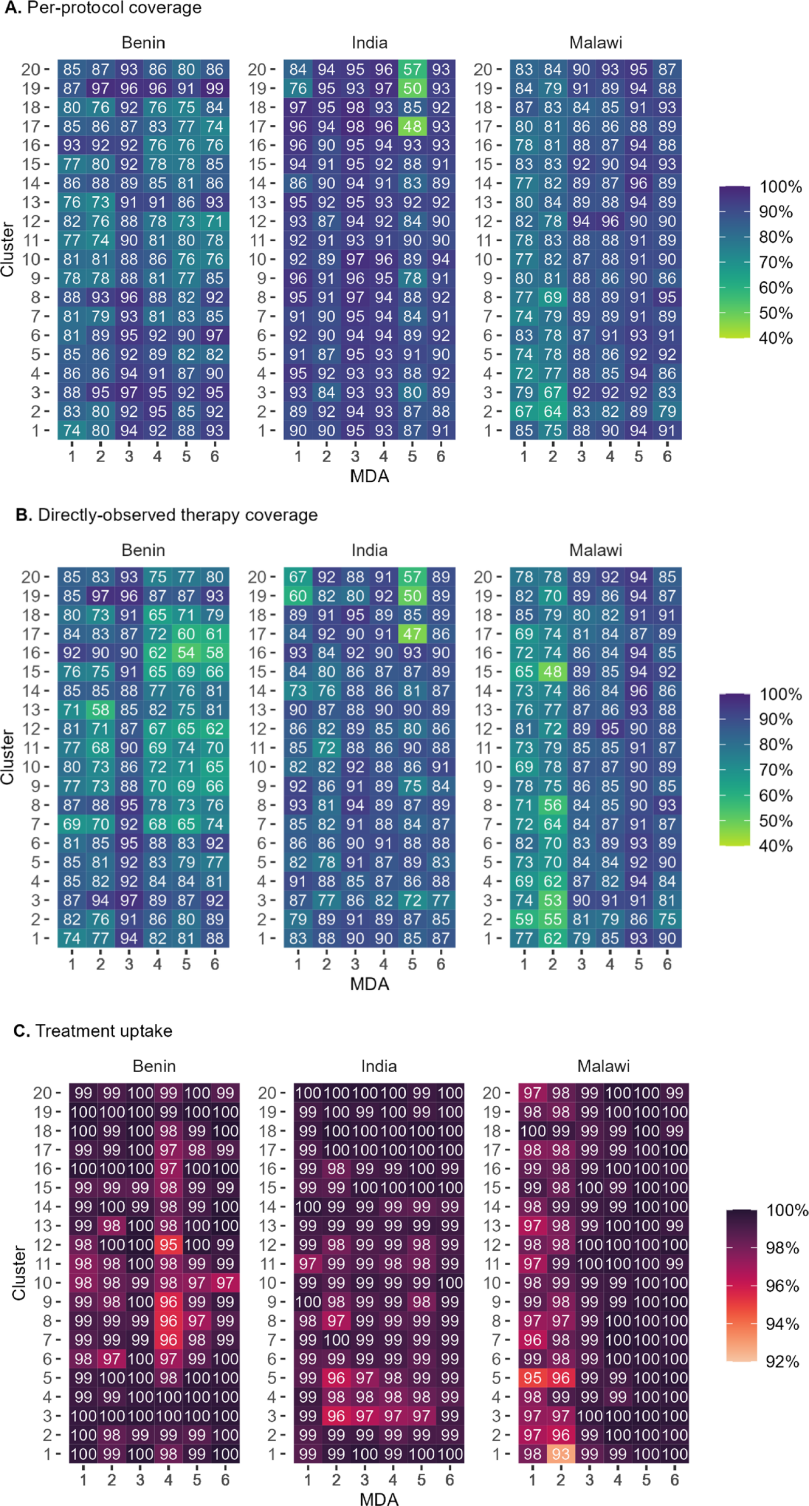
Per-protocol coverage (Panel A), directly observed treatment (DOT) coverage (Panel B), and treatment uptake (Panel C) across the 20 intervention arm clusters at each DeWorm3 site, by cluster and round of mass drug administration (MDA)

Per-protocol coverage was high across all sites and rounds (Fig. 1). In Benin, mean per-protocol coverage at the cluster level ranged from 82% in MDA5 to 92% in MDA3. In Malawi, mean coverage ranged from 78% in MDA2 to 92% in MDA5. In India, mean coverage exceeded 90% (ranging from 91% at MDA2 to 95% at MDA3) at all rounds except MDA5, which was conducted in March 2020 and cut short due to a government COVID-19 lockdown order. Coverage increased over time in many settings (Supplementary Materials 4).

Treatment uptake among eligible individuals reached during MDA exceeded 95% in all clusters across all sites and rounds, with the exception of a single cluster in Malawi during MDA2 where it was 93%. DOT coverage was more varied, ranging from 54% to 97% in Benin, 48% to 96% in Malawi and 47% to 95% in India. DOT coverage was lower at rounds 2, 4, and 6 in Malawi and Benin, as those rounds coincided with treatment of SAC in schools and a smaller number eligible for cMDA, and DOT was consistently higher among children than adults (Fig. 1).

### Cluster-level drivers of coverage

Coverage varied by MDA round at all sites (Table 2). In fully adjusted cluster-level models, coverage was significantly higher in Benin at MDA3 than MDA1; in India, coverage was significantly lower at MDA5 than MDA1; and in Malawi, MDA3-MDA6 all had higher coverage than MDA1. In Benin, proportion speaking a minority language was associated with lower coverage [−2.3% (95% *CI:* −0.5, −4.1%) per category], and number of radio announcements with higher coverage [3.3% (95% *CI:* 0.7, 5.8%) per quartile]. In contrast, in India, the proportion speaking a minority language was associated with higher coverage [1.2% (95% *CI:* 0.1, 2.3%) per category].

**Table 2:** Predictors of per-protocol MDA coverage in DeWorm3 at the cluster level, by site

	Benin (*n* = 120 observations)^a^		India (*n* = 116 observations)^a^		Malawi (*n* = 120 observations)^a^	
Predictors	Adj. dy/dx (95% *CI*)^b^	*P*-value	Adj. dy/dx (95% *CI*)^b^	*P*-value	Adj. dy/dx (95% *CI*)^b^	*P*-value
**MDA round**						
2	−0.058 (−0.0137, 0.021)	0.15	−0.022 (−0.052, 0.008)	0.14	−0.022 (−0.053, 0.009)	0.16
3	**0.104 (0.080, 0.128)**	** < 0.001**	0.020 (−0.003, 0.043)	0.08	**0.095 (0.063, 0.127)**	** < 0.001**
4	0.028 (−0.011, 0.068)	0.16	0.005 (−0.023, 0.033)	0.73	**0.095 (0.065, 0.124)**	** < 0.001**
5	0.021 (−0.028, 0.071)	0.39	**−0.058 (−0.109, −0.008)**	**0.02**	**0.131 (0.095, 0.166)**	** < 0.001**
6	0.010 (−0.034, 0.055)	0.66	−0.007 (−0.057, 0.043)	0.78	**0.103 (0.074, 0.133)**	** < 0.001**
**Proportion of population in cluster**						
Adult (over 15 years), per tertile	0.004 (−0.016, 0.025)	0.67	−0.008 (−0.025, 0.009)	0.37	−0.003 (−0.020, 0.015)	0.76
Male	−0.001 (−0.011, 0.010)	0.91	−0.002 (−0.012, 0.008)	0.66	−0.007 (−0.015, 0.002)	0.12
Migratory, per quintile	−0.002 (−0.017, 0.012)	0.77	0.001 (−0.009, 0.011)	0.90	0.003 (−0.011, 0.016)	0.71
Speaking minority languages^c^	−**0.023 (**−**0.041, **−**0.005)**	**0.01**	**0.012 (0.001, 0.023)**	**0.04**	−0.010 (−0.027, 0.007)	0.24
Minority religion^d^	0.012 (−0.016, 0.040)	0.40	−0.003 (−0.014, 0.009)	0.66	0.003 (−0.015, 0.021)	0.72
Polygamous^e^	0.002 (−0.025, 0.029)	0.89	NA		−0.007 (−0.021, 0.008)	0.37
Cluster mean population density^f^	0.00002 (−0.001, 0.001)	0.96	−0.0002 (−0.001, 0.0003)	0.46	−0.001 (−0.003, 0.001)	0.59
Number of CDDs trained^g^	0.007 (−0.010, 0.024)	0.41	−0.001 (−0.004, 0.002)	0.47	NA	
**Sensitization activities**						
Community meetings	−0.017 (−0.034, 0.001)	0.06	0.001 (−0.001, 0.003)	0.20	0.004 (−0.002, 0.009)	0.20
Public dialogue event	0.003 (−0.001, 0.006)	0.17	Dropped		−0.0004 (−0.004, 0.003)	0.81
Printed IEC materials, quartiles	−0.019 (−0.043, 0.006)	0.14	−0.002 (−0.007, 0.003)	0.38	0.005 (−0.013, 0.024)	0.59
Door to door visits, quartiles	−0.003 (−0.012, 0.005)	0.48	−0.001 (−0.009, 0.008)	0.90	0.006 (−0.002, 0.013)	0.14
Radio, quartiles	**0.033 (0.007, 0.058)**	**0.01**	No activity		No activity	
Other mass media	No activity		Dropped		No activity	

Models used generalized estimating equations with binomial distribution, autoregressive correlation structure and robust standard errors. Adjusted models were selected using the quasi-likelihood under the independence model criterion (QIC). Differences in per-protocol coverage are reported per one unit increase in each predictor (dy/dx). Factors found to be significant at the 0.05 level in the fully adjusted model are indicated in bold

^a^Number of observations per site reflect 20 intervention clusters per site multiplied by six MDA rounds. Four clusters in MDA round 5 were omitted from the India data due to COVID-19 related government shutdown orders

^b^Difference in proportion treated per one unit increase

^c^Three categories in Benin and Malawi, four in India. Majority language: Pedah, Sahoue, Watchi, Mina, Adja, or Xwla in Benin; Chiyao in Malawi; Tamil in India. Minority language: Fon or other in Benin; Chichewa or other in Malawi; Telegu, Urdu, Hindi or other in India

^d^Majority religion: Christianity in Benin; Islam in Malawi; Hinduism in India. Minority religion: Islam, Voodoo, or traditional religion in Benin; Christianity or other in Malawi; Christianity, Islam, or other in India

^e^Not assessed in India. Variable is grouped as “greater than median” or “less than or equal to median”

^f^Mean population density within 0.5 km of households, per 100 people/km^2^

^g^Data not available for all MDA rounds in Malawi

Unadjusted models are available in Supplementary Materials 5.

### Individual-level correlates of non-treatment amongst adults

In fully mutually adjusted models, odds of non-treatment were higher among younger adults than those 50 and older, with the exception of 40–49 year olds in India [odds ratio (*OR*) = 0.77, 95% *CI:* 0.71, 0.83, Table 3]. Non-treatment was highest among those 20–29 years old at all three sites (*OR* = 2.44, 95% *CI:* 2.26, 2.64 in Benin; *OR* = 1.74, 95% *CI:* 1.62, 1.88 in India; and *OR* = 4.25, 95% *CI:* 3.93, 4.59 in Malawi). While there was no significant difference by gender in India, non-treatment was lower among women in Benin (*OR* = 0.83, 95% *CI:* 0.79, 0.88) and in Malawi (*OR* = 0.22, 95% *CI:* 0.21, 0.24). Migration was associated with non-treatment at all three sites, ranging from 3.99-fold odds in Benin (95% *CI:* 3.73, 4.27) to 6.34-fold (95% *CI:* 5.93, 6.79) in Malawi. Compared to unmarried adults, monogamously married adults were less likely to be untreated in India and Benin (*OR* = 0.62, 95% *CI:* 0.59, 0.66; and *OR* = 0.79, 95% *CI:* 0.75, 0.84, respectively), while polygamous marriage was associated with lower odds of non-treatment in Benin (*OR* = 0.71, 95% *CI:* 0.60, 0.83), but higher in Malawi (*OR* = 1.21, 95% *CI:* 1.10, 1.33). Compared to adults with less than primary school education, odds of non-treatment were lower among those with primary school education in India (*OR* = 0.92, 95% *CI:* 0.86, 0.98). Otherwise, education was positively associated with non-treatment, particularly in Malawi where tertiary education was associated with three-fold odds of non-treatment (*OR* = 3.06, 95% *CI:* 1.90, 4.91). Individuals with missing or unknown information on marital status and education were more likely to be untreated than those with complete information.

**Table 3:** Individual-level correlates of non-treatment amongst adults in DeWorm3 intervention clusters

	Benin (*n* = 165,058, 15 + years of age)		India (*n* = 298,237, 20 + years of age)		Malawi (*n *= 153,970, 20 + years of age)	
Predictors	aOR (95% *CI*)	*P*-value	aOR (95% *CI*)	*P*-value	aOR (95% *CI*)	*P*-value
MDA round^a^						
MDA1	1.0		1.0		1.0	
MDA2	0.95 (0.85, 1.05)	0.282	1.26 (1.12, 1.42)	** < 0.001**	1.18 (1.10, 1.26)	** < 0.001**
MDA3	0.44 (0.36, 0.53)	** < 0.001**	0.54 (0.44, 0.67)	** < 0.001**	0.52 (0.47, 0.58)	** < 0.001**
MDA4	0.88 (0.67, 1.15)	0.335	0.71 (0.52, 0.97)	**0.032**	0.52 (0.46, 0.59)	** < 0.001**
MDA5	1.02 (0.71, 1.45)	0.927	1.69 (1.11, 2.57)	**0.014**	0.26 (0.22, 0.31)	** < 0.001**
MDA6	0.60 (0.39, 0.94)	**0.025**	0.78 (0.46, 1.31)	0.351	0.35 (0.29, 0.43)	** < 0.001**
Individual factors						
Age						
15–19 years	1.78 (1.63, 1.95)	** < 0.001**	–		–	
20–29 years	2.44 (2.26, 2.64)	** < 0.001**	1.74 (1.62, 1.88)	** < 0.001**	4.25 (3.93, 4.59)	** < 0.001**
30–39 years	1.92 (1.77, 2.08)	** < 0.001**	1.19 (1.11, 1.28)	** < 0.001**	2.52 (2.33, 2.74)	** < 0.001**
40–49 years	1.44 (1.32, 1.57)	** < 0.001**	0.77 (0.71, 0.83)	** < 0.001**	1.45 (1.32, 1.58)	** < 0.001**
50 + years	1.0		1.0		1.0	
Sex						
Female	0.83 (0.79, 0.88)	** < 0.001**	1.03 (0.98, 1.08)	0.251	0.22 (0.21, 0.24)	** < 0.001**
Male	1.0		1.0		1.0	
Migratory status						
Migratory^b^	3.99 (3.73, 4.27)	** < 0.001**	5.12 (4.79, 5.46)	** < 0.001**	6.34 (5.93, 6.79)	** < 0.001**
Non-migratory	1.0		1.0		1.0	
Marital status						
Unmarried	1.0		1.0		1.0	
Married	0.79 (0.75, 0.84)	** < 0.001**	0.62 (0.59, 0.66)	** < 0.001**	0.96 (0.90, 1.01)	0.136
Married (polygamous) ^c^	0.71 (0.60, 0.83)	** < 0.001**	NA^c^		1.21 (1.10, 1.33)	** < 0.001**
Marital status unknown	2.59 (2.07, 3.23)	** < 0.001**	3.68 (1.77, 7.68)	**0.001**	2.43 (1.73, 3.40)	** < 0.001**
Education level						
No primary education	1.0		1.0		1.0	
Any primary or middle school education	1.05 (0.99, 1.11)	0.091	0.92 (0.86, 0.98)	**0.007**	1.14 (1.08, 1.20)	** < 0.001**
Any secondary or higher secondary education	0.98 (0.92, 1.04)	0.442	1.30 (1.21, 1.40)	** < 0.001**	1.35 (1.23, 1.49)	** < 0.001**
Any higher/tertiary education	1.19 (1.10, 1.29)	** < 0.001**	1.77 (1.62, 1.94)	** < 0.001**	3.06 (1.90, 4.91)	** < 0.001**
Other education level	1.28 (0.95, 1.72)	0.103	2.07 (1.20, 3.56)	**0.009**	1.29 (0.94, 1.78)	0.117
Education level unknown	1.20 (0.94, 1.52)	0.139	3.14 (1.57, 6.26)	**0.001**	2.69 (2.30, 3.14)	** < 0.001**
Household factors						
Wealth quintile						
Lowest quintile	1.0		1.0		1.0	
Low quintile	1.07 (0.99, 1.16)	0.101	0.80 (0.74, 0.88)	** < 0.001**	0.98 (0.90, 1.07)	0.639
Medium quintile	0.89 (0.83, 0.97)	**0.007**	0.79 (0.72, 0.86)	** < 0.001**	0.91 (0.84, 1.00)	**0.043**
High quintile	0.89 (0.82, 0.96)	**0.004**	0.73 (0.66, 0.79)	** < 0.001**	0.95 (0.87, 1.03)	0.237
Highest quintile	0.80 (0.74, 0.87)	** < 0.001**	0.79 (0.72, 0.87)	** < 0.001**	0.95 (0.87, 1.03)	0.230
Household language						
Minority language	1.48 (1.38, 1.60)	** < 0.001**	1.49 (1.30, 1.70)	** < 0.001**	1.49 (1.27, 1.74)	** < 0.001**
Majority language^d^	1.0		1.0		1.0	
Household religion						
Minority religion	0.96 (0.91, 1.01)	0.148	1.12 (0.98, 1.28)	0.085	0.96 (0.84, 1.11)	0.607
Majority religion^e^	1.0		1.0		1.0	
Household population density (1000 population per half km)	0.96 (0.94, 0.98)	** < 0.001**	1.02 (1.00, 1.04)	**0.015**	1.01 (0.98, 1.04)	0.523

Factors associated with non-treatment were assessed using a mixed effects model with random intercepts for cluster and individual and random slopes to account for individual trends over six rounds of MDA. The final adjusted model includes mutual adjustment for all a priori specified variables. Factors found to be significant at the 0.05 level in the fully adjusted modell are indicated in bold

*aOR* adjusted odds ratio, * CI* 95% confidence interval, *MDA* mass drug administration, *N/A* not applicable

^a^MDA5 was interrupted by COVID-19 lockdown orders in India

^b^Defined as living in the household < 6 months of the year in the previous year

^c^Not assessed in India

^d^Majority language is defined as Pedah, Sahoue, Watchi, Mina, Adja, and Xwla in Benin, Tamil in India, and Chiyao in Malawi

^e^Majority religion is defined as Christianity in Benin, Hinduism in India and Islam in Malawi

Odds of non-treatment were lower among adults from wealthier households in Benin and India, with 11–20% lower odds for the three highest wealth quintiles compared to the lowest in Benin, and 20–27% lower odds for all four quintiles compared to the lowest in India. Living in a minority language speaking household was associated with 48–49% greater odds of non-treatment at all three sites; in contrast, no association was found with minority religion. Population density within 0.5 km of the household was associated with reduced odds of non-treatment in Benin, but increased odds in India (*OR* = 0.98, 95% *CI:* 0.96, 0.99; and *OR* = 1.02, 95% *CI:* 1.00, 1.04 per 1000 individuals/0.5 km radius, respectively).

### Individual-level correlates of non-treatment amongst children

In the fully adjusted model, compared to school-attending SAC, school-attending PSAC had higher odds of non-treatment in India (Table 4, *OR* = 1.26, 95% *CI:* 1.09, 1.46) but lower odds in Malawi (*OR* = 0.77, 95% *CI:* 0.70, 0.85), and there was no significant difference in Benin. School-attending young adults were more likely to be untreated in Malawi (*OR* = 1.80, 95% *CI:* 1.68, 1.93) but not in India. However, odds of non-treatment were consistently higher among children in all age categories who were not attending school than school-attending SAC at all three sites, ranging from 18% increased odds for non-school-attending SAC in Benin (*OR* = 1.18, 95% *CI:* 1.10, 1.27) to 3.80-fold odds among non-school-attending young adults in India (*OR* = 3.80, 95% *CI:* 3.36, 4.29).

**Table 4:** Individual-level correlates of non-treatment amongst children in DeWorm3 intervention clusters

	Benin (*n *= 113,383, 1–14 years)		India (*n *= 121,309, 1–19 years)		Malawi (*n* = 202,369, 2–19 years)	
Predictors	aOR (95% *CI*)	*P*-value	aOR (95% *CI*)	*P*-value	aOR (95% *CI*)	*P*-value
MDA round^a^						
MDA1	1.0		1.0		1.0	
MDA2	1.00 (0.88, 1.13)	0.972	0.84 (0.74, 0.96)	**0.011**	1.01 (0.95, 1.07)	0.808
MDA3	0.40 (0.32, 0.50)	** < 0.001**	0.33 (0.27, 0.41)	** < 0.001**	0.38 (0.34, 0.41)	** < 0.001**
MDA4	0.81 (0.59, 1.11)	0.191	0.45 (0.33, 0.61)	** < 0.001**	0.42 (0.37, 0.47)	** < 0.001**
MDA5	1.00 (0.66, 1.52)	0.990	1.11 (0.74, 1.67)	0.603	0.21 (0.18, 0.25)	** < 0.001**
MDA6	0.58 (0.35, 0.97)	**0.040**	0.59 (0.36, 0.97)	**0.039**	0.29 (0.24, 0.36)	** < 0.001**
Individual factors						
Age category and school attendance^b^						
PSAC (1–4 years) currently attending school	1.09 (0.95, 1.26)	0.231	1.26 (1.09, 1.46)	**0.002**	0.77 (0.70, 0.85)	** < 0.001**
PSAC (1–4 years) not currently attending school	1.18 (1.10, 1.27)	** < 0.001**	2.17 (1.93, 2.43)	** < 0.001**	1.11 (1.04, 1.19)	**0.002**
SAC (5–14 years) currently attending school	1.0		1.0		1.0	
SAC (5–14 years) not currently attending school	1.51 (1.39, 1.65)	** < 0.001**	1.83 (1.51, 2.22)	** < 0.001**	1.47 (1.37, 1.58)	** < 0.001**
Young adults (15–19 years) currently attending school	*NA*^b^	–	1.09 (0.97, 1.21)	0.147	1.80 (1.68, 1.93)	** < 0.001**
Young adults currently not attending or completed school	*NA*^b^	–	3.80 (3.36, 4.29)	** < 0.001**	3.14 (2.92, 3.38)	** < 0.001**
Sex						
Male	1.0		1.0		1.0	
Female	1.14 (1.07, 1.22)	** < 0.001**	1.13 (1.04, 1.24)	**0.005**	1.09 (1.03, 1.14)	**0.001**
Migratory status						
Migratory^c^	4.87 (4.38, 5.41)	** < 0.001**	5.20 (4.64, 5.83)	** < 0.001**	6.68 (6.11, 7.31)	** < 0.001**
Non-migratory	1.0		1.0		1.0	
Household factors						
Wealth quintile						
Lowest quintile	1.0		1.0		1.0	
Low quintile	0.97 (0.87, 1.09)	0.631	0.86 (0.74, 1.00)	**0.046**	0.80 (0.74, 0.87)	** < 0.001**
Medium quintile	0.80 (0.72, 0.89)	** < 0.001**	0.81 (0.70, 0.95)	**0.008**	0.76 (0.70, 0.83)	** < 0.001**
High quintile	0.87 (0.78, 0.97)	**0.015**	0.73 (0.63, 0.85)	** < 0.001**	0.81 (0.75, 0.88)	** < 0.001**
Highest quintile	0.88 (0.78, 0.99)	**0.027**	0.77 (0.66, 0.91)	**0.002**	0.95 (0.87, 1.03)	0.213
Household language						
Minority language	1.80 (1.62, 2.00)	** < 0.001**	1.56 (1.18, 2.07)	**0.002**	2.32 (1.99, 2.72)	** < 0.001**
Majority language^d^	1.0		1.0		1.0	
Household religion						
Minority religion	0.81 (0.75, 0.87)	** < 0.001**	1.31 (1.03, 1.67)	**0.031**	0.93 (0.80, 1.07)	0.279
Majority religion^e^	1.0		1.0		1.0	
Highest household resident education level						
No primary education	1.0		1.0		1.0	
Any primary or middle school education	0.99 (0.91, 1.08)	0.782	0.68 (0.58, 0.80)	** < 0.001**	0.88 (0.83, 0.94)	** < 0.001**
Any secondary or higher secondary education	0.88 (0.81, 0.95)	**0.001**	0.64 (0.54, 0.75)	** < 0.001**	1.00 (0.92, 1.08)	0.958
Any higher/tertiary education	1.00 (0.91, 1.11)	0.942	0.94 (0.79, 1.12)	0.500	1.52 (1.10, 2.10)	**0.012**
Other education level	2.18 (1.20, 3.93)	**0.010**	–	–	1.97 (0.91, 4.28)	0.086
Education level unknown	6.08 (3.33, 11.08)	** < 0.001**	–	–	5.27 (3.79, 7.32)	** < 0.001**
Population density within 0.5 km of the household (per 1000 population)	1.04 (1.02, 1.05)	** < 0.001**	1.03 (1.00, 1.07)	**0.047**	1.03 (1.00, 1.06)	**0.026**

Factors associated with non-treatment were assessed using a mixed effects model with random intercepts for cluster and individual and random slopes to account for individual trends over six rounds of MDA. The final adjusted model includes mutual adjustment for all a priori specified variables. Factors found to be significant at the 0.05 level in the fully adjusted model are indicated in bold

*aOR* adjusted odds ratio, 95% *CI* 95% confidence interval, *PSAC* pre-school-age children, *SAC* school-age children, *NA* not applicable

^a^MDA5 was interrupted by COVID-19 lockdown orders in India

^b^Young adults 15–19 years not eligible for standard-of-care deworming in Benin and not included in the pediatric model

^c^Defined as living in the household < 6 months in the previous year

^d^Majority language is defined as Pedah, Sahoue, Watchi, Mina, Adja, and Xwla in Benin, Tamil in India, and Chiyao in Malawi

^e^Majority religion is defined as Christianity in Benin, Hinduism in India and Islam in Malawi

Girls had consistently increased odds of non-treatment compared to boys at all three sites (Benin: *OR* = 1.14, 95% *CI:* 1.07, 1.22; India: *OR* = 1.13, 95% *CI:* 1.04, 1.24; Malawi: *OR* = 1.09, 95% *CI:* 1.03, 1.14).

Of factors potentially associated with treatment access, migration had the strongest association with non-treatment, with 4.87-fold odds in Benin (95% *CI:* 4.38, 5.41), 5.20-fold in India (95% *CI:* 4.64, 5.83), and 6.68-fold in Malawi (95% *CI:* 6.11, 7.31). In general, increased household wealth was associated with decreased odds of non-treatment, though there was not a dose-dependent trend. Compared to the lowest wealth quintile, children from the highest three quintiles in Benin had 12–20% decreased odds of non-treatment, in India all four quintiles had 14–27% decreased odds, and in Malawi the second, third and fourth had 19–24% decreased odds. Children from households speaking minority languages had substantially greater odds of non-treatment at all three sites, 80% in Benin (*OR* = 1.80, 95% *CI:* 1.62, 2.00), 56% in India (*OR* = 1.56, 95% *CI:* 1.18, 2.07), and 2.32-fold in Malawi (*OR* = 2.32, 95% *CI:* 1.99, 2.72). In contrast, children belonging to minority religion households had decreased odds of being untreated in Benin (*OR* = 0.81, 95% *CI:* 0.75, 0.87) but increased odds in India (*OR* = 1.31, 95% *CI:* 1.03, 1.67), and no difference compared to majority religion households in Malawi.

In contrast to adults’ own treatment, children’s treatment was positively associated with adult education in the household. Compared to children in households where adults had no primary school education, children living with adults with a primary or middle school education in India and Malawi were less likely to be untreated (*OR* = 0.68, 95% *CI:* 0.58, 0.80; and *OR* = 0.88, 95% *CI:* 0.83, 0.94), as were those living with adults with a secondary education in Benin and India (*OR* = 0.88, 95% *CI:* 0.81, 0.95; and *OR* = 0.64, 95% *CI:* 0.54, 0.75, respectively). Unknown adult education level was associated with increased odds of non-treatment in Benin and Malawi (*OR* = 6.08, 95% *CI:* 3.33, 11.08; and *OR* = 5.27, 95% *CI:* 3.79, 7.32).

Population density was associated with increased odds of being untreated at all three sites; 4% per 1000 residents within 0.5 km in Benin (*OR* = 1.04, 95% *CI:* 1.03, 1.05), and 3% in India (*OR* = 1.03, 95% *CI: *1.00, 1.07) and Malawi (*OR* = 1.03, 95% *CI:* 1.00, 1.06).

## Discussion

The DeWorm3 study achieved high coverage across all three sites, consistently exceeding the WHO “NTD Roadmap” target of treating 75% of PSAC and SAC to control STH-associated morbidities [27]. Prior modeling suggests that it may be feasible to interrupt STH transmission in high transmission settings if coverage of 80–90% of all age groups is attained, though targeted levels vary depending on dominant STH species and human migration patterns [11].

Treatment uptake was extremely high among people successfully reached by MDA, exceeding 95% in nearly all treatment rounds. However, DOT was less acceptable, particularly among adults, and especially in Malawi and Benin. This finding converges with qualitative data in DeWorm3 sites that DOT was viewed more favorably in clusters with higher coverage and may have actually been a deterrent to accepting treatment in clusters with lower coverage [28]. Of factors assessed as potential proxies for marginalization within study sites, migration was most strongly associated with non-treatment, which is partly explained by migratory individuals being more likely to be absent or harder to locate during cMDA.

The observation that girls were less likely to be treated than boys at all three sites, in contrast with adult women who were more likely to be treated in two sites, is concerning, as prior analysis of routine program data across MDA platforms suggested that there may not be major gaps in coverage equity by gender [29]. However, gender disaggregated treatment data are rarely available at national or global levels [30]. Qualitative findings from DeWorm3 study sites indicate that women in lower coverage clusters exhibited less decision-making latitude on behalf of their households as compared to women in higher coverage clusters [28]. Deliberate engagement of women in cMDA activities may serve to increase coverage and may also have gender transformative effects.

Odds of non-treatment were highest among individuals from the poorest households at each site, who were also those most likely to be infected with hookworm at the start of the trial [31–33]. Children and young adults who did not or never attended school were consistently more likely to be untreated, despite the community-based treatment approach. In contrast, highly educated adults were more likely to deworm their children but be untreated themselves, perhaps perceiving themselves to be at lower risk. Speakers of minority languages were substantially more likely to be untreated. Notably individuals with “missing information” were frequently untreated, indicating that missing census data may be a proxy for an individual being hard to reach or not trusting of the research teams and potentially marginalized. Each of these risk factors is likely driven by unique social conditions, including cultural beliefs and trust in the healthcare system, and many were identified by community members in DeWorm3 study areas prior to the launch of the study [34].

It is well established that multi-level sensitization of community members and local leaders is important to achieve high coverage of MDA or other community-based public health activities [35]. In this study, radio announcements were the only cluster-level sensitization activity significantly associated with coverage. However, associations between cluster-level coverage and DeWorm3 sensitization activities are challenging to interpret. Sensitization efforts were tailored between rounds, including launching more intensive sensitization in clusters where coverage was previously low, reducing our ability to detect the effect of specific sensitization activities above and beyond that explained by trends over MDA rounds. Tailored sensitization activities could be further applied to address observed correlates of coverage, such as specifically addressing minority language speakers, and targeting highly educated households with messaging that might be more likely to influence behaviors (e.g., via social media) despite a potentially lower perceived risk of STH infection.

Strengths of the current study include the use of censuses to accurately determine the target population for deworming, and real-time data collection on individual-level treatment status. Limitations include possible misclassification of the treatment status of individuals who were not directly reached by drug distributors, but whose tablets were left at their households on the third visit. While the DeWorm3 project demonstrated that high coverage of community-wide deworming can be achieved across diverse settings when there is sufficient personnel to conduct intensive planning and real-time decision making, the limited resources available to support MDA activities in many settings may preclude generalizability. The high coverage observed does not indicate that transmission interruption is inevitable in DeWorm3 sites, rather that if transmission interruption is feasible, DeWorm3 sites may provide optimal conditions to observe it.

## Conclusions

This study demonstrates that it is possible to implement cMDA with high treatment coverage, and to improve coverage over time. Despite the high coverage observed, some communities and individuals remain at higher risk of not being treated, including girls, migrants, minority language speakers, children and young adults not attending schools, individuals of lower wealth status and, in some cases, those living in more densely populated areas. Most of these factors are consistent across the very heterogenous DeWorm3 settings, indicating that tailored strategies to address these factors may have significant impact on coverage across NTD endemic areas.

## Supplementary Information

Additional file 1

Additional file 2

Additional file 3

Additional file 4

Additional file 5

## Data Availability

All of the individual participant data and cluster-level data that underlie the results reported in this article will be shared after de-identification and within one year following publication. Data will be open access and available for any purpose. In the short-term, proposals for data use can be emailed directly to the corresponding author.
