# Supplementary material for: Coverage of community-wide mass drug administration platforms for soil-transmitted helminths in Benin, India, and Malawi: findings from the DeWorm3 project
Source: Infect Dis Poverty. 2024 Oct 8;13:72. doi: 10.1186/s40249-024-01241-0 (PMC11460046; doi:10.1186/s40249-024-01241-0)
Supplement: Supplementary file 1 — Additional file 1 [file 40249_2024_1241_MOESM1_ESM.docx]

**Supplementary materials**

# **S1. Additional details on DeWorm3 activities implemented**

In the below tables, we provide additional details on how community-wide mass drug administration (cMDA) and school-based deworming (SBD) were implemented in each country.

## **S1. Table 1: Implementation characteristics of the DeWorm3 trial at study sites**

|  | Benin | India | Malawi |
| --- | --- | --- | --- |
| Study location | Come Commune | Tamil Nadu State (Vellore and Thiruvanamalai districts) | Mangochi District |
| Implementing organizations | - Institut de Recherche Clinique du Benin - Institut de Recherche pour le Développement - Ministry of Health, Benin | - Christian Medical College, Vellore - Ministry of Health and Family Welfare, New Delhi and Directorate of Public Health, Chennai | - Blantyre Institute for Community Outreach - London School of Tropical Medicine and Hygiene - Ministry of Health and Education, Malawi |
| cMDA strategy (20 intervention clusters) | - Bi-annual cMDA in all ages. - Community drug distributors (volunteers) delivered drugs - Implemented by DeWorm3 | - Bi-annual cMDA in all ages; following National Deworming Day (described below) - Community drug distributors (volunteers) delivered drugs - Implemented by DeWorm3 | - Bi-annual cMDA in all ages - Health Surveillance Assistants, employed by the government, delivered drugs - Implemented by DeWorm3 |
| SBD strategy (20 intervention clusters and 20 control clusters) | - SBD conducted annually - Treatment of children 5-14 years old - Implemented by the Ministry of Health (with a subcontract from DeWorm3) | - National Deworming Days, conducted bi-annually in schools and Anganwadi centers (pre-schools) - Treatment of children 1-19 years old - Implemented by the Ministry of Health and Family Welfare, New Delhi and Directorate of Public Health, Chennai | - SBD conducted annually, integrated with “Child Health Days” - Community mop-up for non-enrolled children - Treatment of children 1-14 years old - Implemented by DeWorm3 |
| Additional trial activities | - Planning meetings - Annual census - Two prevalence surveys - Bi-annual coverage survey (after each round of MDA) | - Planning meetings - Annual census - Two prevalence surveys - Bi-annual coverage survey (after each round of MDA) | - Planning meetings - Annual census - One prevalence survey - Bi-annual coverage survey (after each round of MDA) |

Acronyms: mass drug administration (MDA), community-wide MDA (cMDA), school-based deworming (SBD).

## **S1. Table 2: Narrative description of DeWorm3 community-wide mass drug administration activities**

| **Sub-activity** | **Benin** | **Malawi** | **India** |
| --- | --- | --- | --- |
| Supply chain | - *Shipment to country*: Drugs were donated, ordered through the WHO. Drugs were ordered centrally by the Ministry of Health, for routine use, and stored in the national storage facility. - *Storage and disbursement:* Drugs were dispatched to the zonal referral hospital in Come, by the National Communicable Disease Control Program (Programme National de Lutte contre les Maladies Transmissibles or PNLMT). Afterward, drugs were transferred to each health center affiliated with DeWorm3, with transit supervised by head doctors at the commune level. Nurses collected drugs for the MDA campaign from the referral hospital after training. Nurses then dispensed drugs to CDDs for cMDA. After cMDA, the remaining drugs were transported from clusters to the central level. | - *Shipment to country*: Drugs were donated, ordered through the WHO. One shipment for both cMDA and SBD was made for 1.5 million doses and sent by ship, which supplied all years of the project. The stock was kept at the Central Drug Stores in Lilongwe and then dispensed to the study. - *Storage and disbursement*: Albendazole for each MDA round was stored in the Deworm3 office in Namwera. During cMDA, albendazole was dispensed daily to the enumerators, and the remaining stock was returned to the office each evening. Drug supply was monitored using stock control cards and excel files of stock issued to enumerators. | - *Shipment to country*: Drugs were donated, ordered through the WHO. Drugs were ordered centrally by the Ministry of Health and Family Welfare, through the national NTD program. - *Storage and disbursement*: Consignment was brought to the central DeWorm3 office in Vellore and subsequently delivered to two subsite field offices. DeWorm3 field supervisors managed the tablets and provided them to fieldworkers daily to take to the villages for community drug distributors (CDDs) to dispense. The remaining tablets were returned to the office at end of the day. |
| Sensitization | - Information sessions were held with local authorities (town hall), leaders of opinion, religious leaders, professional associations, and town criers. - Messages were passed to the community through town criers, radio broadcasts, specific groups (i.e. women’s associations), and religious centers. - Banners and posters were also placed in the community. | Several committees and community boards were engaged for MDA sensitization. In year 2, the DeWorm3 team employed additional sensitization measures to improve community engagement and maximize treatment coverage. Activities included:   - Area Development Council meetings with group village headmen and/or representatives from Village Development Committees. - Village-level community meetings were conducted by Health Surveillance Associates (HSAs) and volunteers. - Village dramas and public announcements (year 2 only). - Religious and Traditional Authority leaders of the Community Advisory Board visited communities that displayed signs of community tension or low participation to resolve any communication issues (year 2 only). | - National Deworming Day sensitization materials were adapted to include information on cMDA; 1000 posters and 200 banners were posted in villages. - Community sensitization meetings were conducted by DeWorm3 field staff using locally designed flipbooks to explain how STH are transmitted and what activities would be undertaken during cMDA. |
| Training | - *Health staff and volunteers:* Ministry of Health staff trained 10 head health personnel (health center nurses, Chief Medical Officer, and District Medical Coordinator). Head nurses then trained CDDs. Supervision of training was done by PNLMT technical staff, doctors, and some district and departmental level staff. - *Enumerators:* DeWorm3 staff trained enumerators and controllers (supervisors of enumerators). | - *Health staff and volunteers*: DeWorm3 field officers trained HSAs at health centers and halls. Training sessions were one day long, though they were conducted over the course of two days to accommodate all health center staff. Afterward, HSAs oriented volunteers. - *Enumerators:* The DeWorm3 trial coordinator and field officers trained enumerators for two days, followed by a three-day pilot of data collection instruments used during MDA. | - *Health staff and volunteers*: CDDs participated in a half-day training, conducted by the DeWorm3 medical officer. - *Enumerators:* DeWorm3 fieldworkers were trained by the DeWorm3 trial coordinator and data manager, followed by a short pilot period to test forms used during MDA. |
| Drug delivery | Drug delivery was conducted twice per year, in intervention clusters only (n=20). Drugs were distributed by CDDs, joined by an enumerator, with the assumption that each CDD/enumerator pair would treat 60 people per day. | Drug delivery was conducted twice per year, in intervention clusters only (n=20), by teams of enumerators, HSAs, and volunteers. HSAs were responsible for a relatively large number of households. HSAs supervised volunteers (about 4 volunteers per HSA). Enumerators were driven daily from Namwera to the community with their drug stocks, and HSAs were picked up along the way. Area Development Council members helped in mobilizing the community on the day of MDA. | Drug delivery was conducted twice per year, in intervention clusters only (n=20), by teams of DeWorm3 fieldworkers (serving as enumerators) and CDDs, who walked door to door in the community. Nurses and medical officers supported with adverse events. |
| Supervision | Supervision was conducted by DeWorm3 staff, central PNLMT staff, departmental staff, District Chief Doctors, and sub-district health center nurses. | Supervision was conducted by the DeWorm3 trial coordinator, DeWorm3 field officers, local health officers (Environmental Health Officers, Assistant Environmental Health Officers, District Environmental Health Officer, District Health Officer), District Council Representative, District STH Coordinator, and the Ministry of Health STH Programme Manager. | Supervision was conducted by DeWorm3 field supervisors, DeWorm3 field managers, and local health workers (Village Head Nurses, Sector Health Nurses, and Community Health Nurses, and Block Medical Officers). |
| Mop-up | Two days of mop-up was conducted as needed. There was no mop-up in round 1 of cMDA. In round 4, flooding interrupted cMDA, and extensive mop-up was conducted. | Malawi did not have a distinct mop-up period for cMDA. Instead, progress on coverage was tracked by a DeWorm3 monitoring dashboard, informed by electronic data collection forms. MDA was only considered complete once the dashboard indicated that all households had been treated or visited three times; all individuals who were absent from the household, but not migrated, at the first visit were followed up at least two further times. | After cMDA, a mop-up campaign was conducted for 1-4 days to reach absent individuals. Homes with absent individuals were visited up to three times. |

Acronyms: World Health Organization (WHO), mass drug administration (MDA), community-wide mass drug administration (cMDA), school-based deworming (SBD), soil-transmitted helminths (STH), neglected tropical diseases (NTD), Programme National de Lutte contre les Maladies Transmissibles (PNLMT).
