# Supplementary material for: Coverage of community-wide mass drug administration platforms for soil-transmitted helminths in Benin, India, and Malawi: findings from the DeWorm3 project
Source: Infect Dis Poverty. 2024 Oct 8;13:72. doi: 10.1186/s40249-024-01241-0 (PMC11460046; doi:10.1186/s40249-024-01241-0)
Supplement: Supplementary file 2 — Additional file 2 [file 40249_2024_1241_MOESM2_ESM.docx]

|  | **MDA Round** | | | | | |  |
| --- | --- | --- | --- | --- | --- | --- | --- |
| **Sensitization activity** | **1** | **2** | **3** | **4** | **5** | **6** | **Total** |
| **Benin** | N=20 | N=20 | N=20 | N=20 | N=20 | N=20 | N=120 |
| Community meetings | 19 (95.0%) | 20 (100.0%) | 20 (100.0%) | 20 (100.0%) | 20 (100.0%) | 20 (100.0%) | 119 (99.2%) |
| Public dialogue event* | 20 (100.0%) | 20 (100.0%) | 20 (100.0%) | 20 (100.0%) | 20 (100.0%) | 20 (100.0%) | 120 (100.0%) |
| Distribution of printed IEC materials | 20 (100.0%) | 20 (100.0%) | 20 (100.0%) | 20 (100.0%) | 20 (100.0%) | 20 (100.0%) | 120 (100.0%) |
| Door-to-door sensitization | 0 (0.0%) | 0 (0.0%) | 0 (0.0%) | 17 (85.0%) | 20 (100.0%) | 20 (100.0%) | 57 (47.5%) |
| Radio | 0 (0.0%) | 19 (95.0%) | 19 (95.0%) | 20 (100.0%) | 19 (95.0%) | 20 (100.0%) | 97 (80.8%) |
| TV | 0 (0.0%) | 1 (5.0%) | 1 (5.0%) | 0 (0.0%) | 0 (0.0%) | 0 (0.0%) | 2 (1.7%) |
| Newspaper | 0 (0.0%) | 0 (0.0%) | 0 (0.0%) | 0 (0.0%) | 0 (0.0%) | 0 (0.0%) | 0 (0.0%) |
| Other mass media | 0 (0.0%) | 0 (0.0%) | 0 (0.0%) | 0 (0.0%) | 20 (100.0%) | 20 (100.0%) | 40 (33.3%) |
| Number CDDs trained (cluster median and range) | 4 (2-7) | 5 (4-6) | 4 (3-7) | 4 (3-7) | 4 (3-7) | 4 (3-7) | 4 (2-7) |
| **India** | N=20 | N=20 | N=20 | N=20 | N=16 | N=20 | N=116 |
| Community meetings | 15 (75.0%) | 18 (90.0%) | 20 (100.0%) | 20 (100.0%) | 15 (93.8%) | 1 (5.0%) | 89 (76.7%) |
| Public dialogue event* | 0 (0.0%) | 17 (85.0%) | 20 (100.0%) | 20 (100.0%) | 2 (12.5%) | 16 (80.0%) | 75 (64.7%) |
| Distribution of printed IEC materials | 20 (100.0%) | 20 (100.0%) | 17 (85.0%) | 20 (100.0%) | 16 (100.0%) | 20 (100.0%) | 113 (97.4%) |
| Door-to-door sensitization | 0 (0.0%) | 20 (100.0%) | 16 (80.0%) | 19 (95.0%) | 16 (100.0%) | 20 (100.0%) | 91 (78.4%) |
| Radio | 0 (0.0%) | 0 (0.0%) | 0 (0.0%) | 0 (0.0%) | 0 (0.0%) | 0 (0.0%) | 0 (0.0%) |
| TV | 0 (0.0%) | 0 (0.0%) | 0 (0.0%) | 0 (0.0%) | 0 (0.0%) | 0 (0.0%) | 0 (0.0%) |
| Newspaper | 0 (0.0%) | 0 (0.0%) | 0 (0.0%) | 0 (0.0%) | 0 (0.0%) | 0 (0.0%) | 0 (0.0%) |
| Other mass media | 0 (0.0%) | 9 (45.0%) | 0 (0.0%) | 19 (95.0%) | 13 (81.3%) | 1 (5.0%) | 42 (36.2%) |
| Number CDDs trained (cluster median and range) | 8 (4-15) | 3 (0-8) | 3.5 (0-6) | 3 (0-8) | 4 (0-8) | 3 (0-8) | 4 (0-15) |
| **Malawi** | N=20 | N=20 | N=20 | N=20 | N=20 | N=20 | N=120 |
| Community meetings | 19 (95.0%) | 19 (95.0%) | 20 (100.0%) | 20 (100.0%) | 20 (100.0%) | 20 (100.0%) | 118 (98.3%) |
| Public dialogue event* | 1 (5.0%) | 5 (25.0%) | 20 (100.0%) | 20 (100.0%) | 20 (100.0%) | 20 (100.0%) | 86 (71.7%) |
| Distribution of printed IEC materials | 1 (5.0%) | 0 (0.0%) | 0 (0.0%) | 0 (0.0%) | 0 (0.0%) | 0 (0.0%) | 1 (0.8%) |
| Door-to-door sensitization | 3 (15.0%) | 15 (75.0%) | 0 (0.0%) | 0 (0.0%) | 0 (0.0%) | 0 (0.0%) | 18 (15.0%) |
| Radio | 0 (0.0%) | 0 (0.0%) | 0 (0.0%) | 0 (0.0%) | 0 (0.0%) | 0 (0.0%) | 0 (0.0%) |
| TV | 0 (0.0%) | 0 (0.0%) | 0 (0.0%) | 0 (0.0%) | 0 (0.0%) | 0 (0.0%) | 0 (0.0%) |
| Newspaper | 0 (0.0%) | 0 (0.0%) | 0 (0.0%) | 0 (0.0%) | 0 (0.0%) | 0 (0.0%) | 0 (0.0%) |
| Other mass media | 0 (0.0%) | 0 (0.0%) | 0 (0.0%) | 0 (0.0%) | 0 (0.0%) | 0 (0.0%) | 0 (0.0%) |
| Number CDDs trained (cluster median and range) | 1 (1-4) | 1 (1-2) | 60 (6-60) | 8 (6-11) | 7 (6-9) | 0 | 5 (0-60) |

**S2. Sensitization Activities by site and MDA round**

* Including public presentations of information in formats including town criers, community dramas, loudspeakers on motorbikes, etc.
