# Supplementary material for: Coverage of community-wide mass drug administration platforms for soil-transmitted helminths in Benin, India, and Malawi: findings from the DeWorm3 project
Source: Infect Dis Poverty. 2024 Oct 8;13:72. doi: 10.1186/s40249-024-01241-0 (PMC11460046; doi:10.1186/s40249-024-01241-0)
Supplement: Supplementary file 3 — Additional file 3 [file 40249_2024_1241_MOESM3_ESM.docx]

**S3. DeWorm3 definitions of coverage.**

| **Coverage indicator** | **Definition*** |
| --- | --- |
| Coverage definitions generated via MDA treatment logs in intervention clusters (N=20 per site) | |
| Per protocol coverage | Proportion of censused and eligible† individuals treated‡ amongst all censused and eligible individuals |
| Treatment uptake | Proportion of censused and eligible† cluster residents accepting treatment‡ amongst all censused and eligible cluster residents reached during MDA and not recently treated |
| Directly observed treatment (DOT) coverage | Proportion of censused and eligible† individuals receiving DOT amongst all censused and eligible individuals not recently treated |

* All definitions exclude individuals who died since the census, infants <1 year old (<2 years old in Malawi), and pregnant women in the first trimester.

† Individuals are considered censused and eligible if they are alive at the time of MDA, not in their first trimester of pregnancy, residing in the study area, included in the DeWorm3 census, ≥1 year old (Benin and India) or ≥2 years old (Malawi).

‡ Reported treatment with albendazole outside DeWorm3 in the two weeks prior to MDA (“recently treated”), treated via DOT, treated but not via DOT.
