# Supplementary material for: Coverage of community-wide mass drug administration platforms for soil-transmitted helminths in Benin, India, and Malawi: findings from the DeWorm3 project
Source: Infect Dis Poverty. 2024 Oct 8;13:72. doi: 10.1186/s40249-024-01241-0 (PMC11460046; doi:10.1186/s40249-024-01241-0)

**S4. Per-protocol MDA coverage in intervention clusters across all three DeWorm3 sites, by cluster (line) and round of MDA**


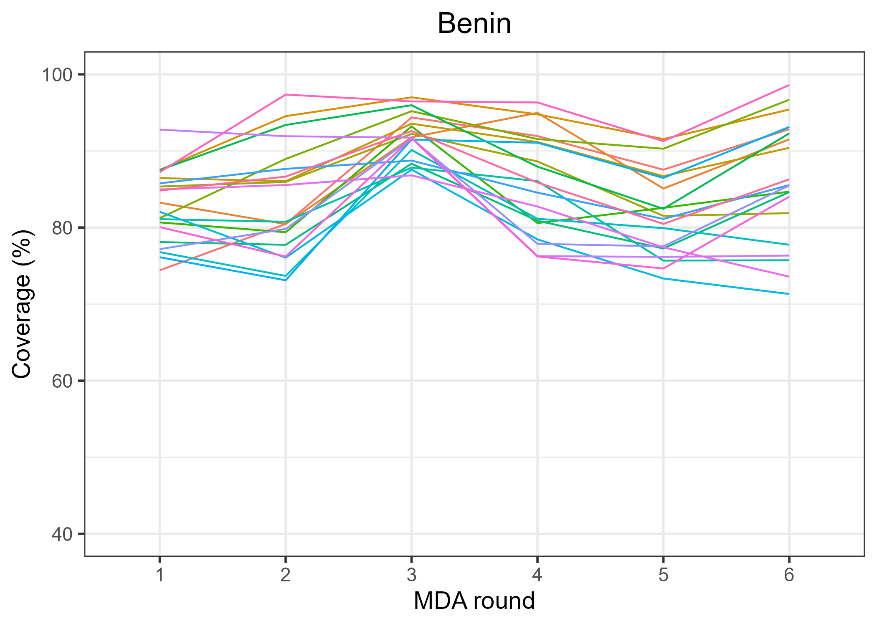

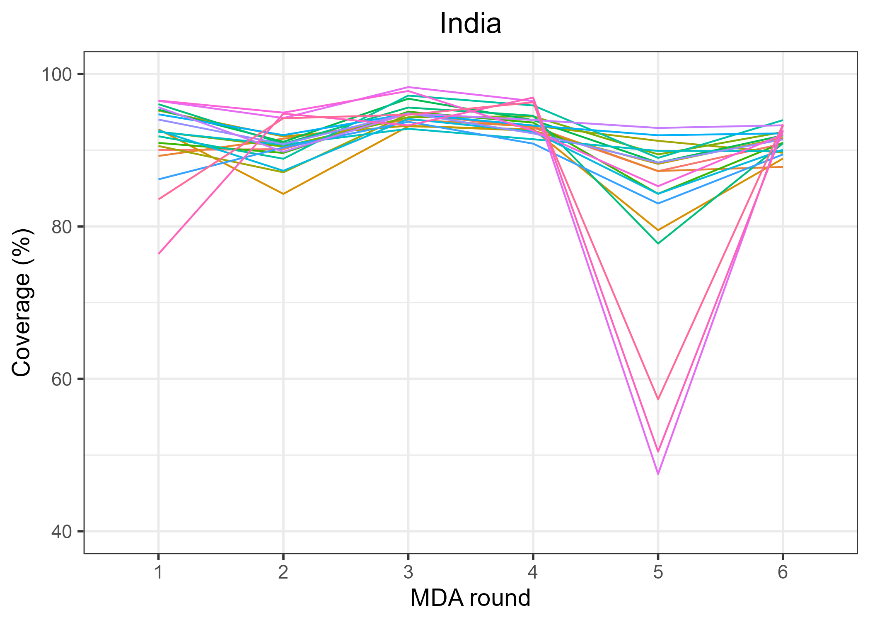

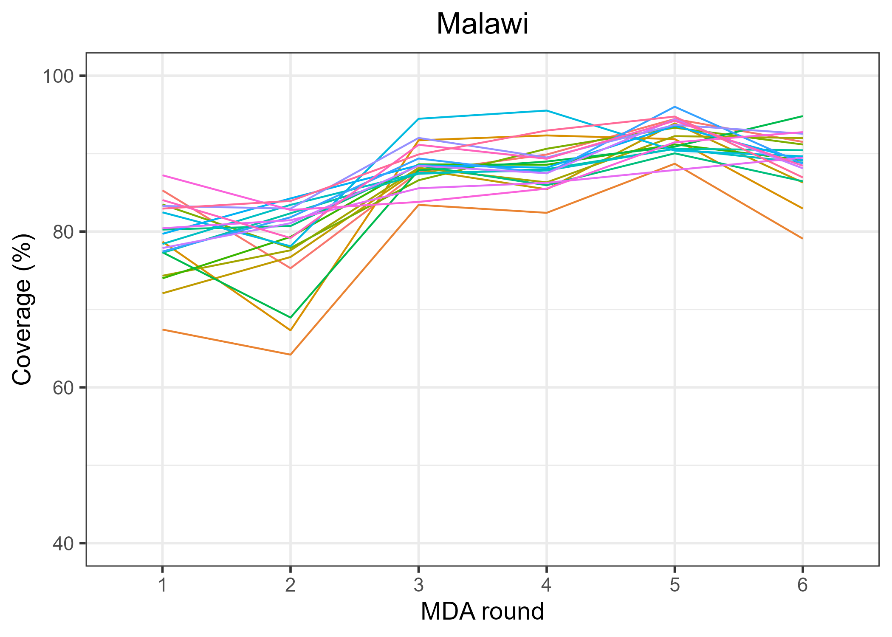

Supplement: Supplementary file 4 — Additional file 4 [file 40249_2024_1241_MOESM4_ESM.docx]
