# Supplementary material for: Coverage of community-wide mass drug administration platforms for soil-transmitted helminths in Benin, India, and Malawi: findings from the DeWorm3 project
Source: Infect Dis Poverty. 2024 Oct 8;13:72. doi: 10.1186/s40249-024-01241-0 (PMC11460046; doi:10.1186/s40249-024-01241-0)
Supplement: Supplementary file 5 — Additional file 5 [file 40249_2024_1241_MOESM5_ESM.docx]

**S5. Unadjusted and adjusted models of predictors at cluster and individual-levels, by site**

**S5. Table 1.** **Predictors of per-protocol MDA coverage in DeWorm3 at the cluster level, by site**

*Models used generalized estimating equations with binomial distribution, autoregressive correlation structure and robust standard errors. Base models are adjusted for MDA round only, while fully adjusted models were selected using the quasi-likelihood under the independence model criterion (QIC). Differences in per-protocol coverage are reported per one unit increase in each predictor (dy/dx).*

|  | **Benin**  **(N = 120 observations)*** | | | | **India**  **(N = 116 observations)*** | | | | **Malawi**  **(N = 120 observations)*** | | | |
| --- | --- | --- | --- | --- | --- | --- | --- | --- | --- | --- | --- | --- |
|  | **Unadjusted** | | **Adjusted** | | **Unadjusted** | | **Adjusted** | | **Unadjusted** | | **Adjusted** | |
| **Predictors** | ***dy/dx (95% CI)***† | ***P-value*** | ***dy/dx (95% CI)***† | ***P-value*** | ***dy/dx (95% CI)***† | ***P-value*** | ***dy/dx (95% CI)***† | ***P-value*** | ***dy/dx (95% CI)***† | ***P-value*** | ***Adj. dy/dx (95% CI)***† | ***P-value*** |
| **MDA round** |  |  |  |  |  |  |  |  |  |  |  |  |
| 2 | 0.012 (-0.005-0.030) | 0.16 | -0.058 (-0.0137-0.021) | 0.15 | -0.009 (-0.035-0.017) | 0.48 | -0.022 (-0.052-0.008) | 0.14 | -0.008 (-0.030-0.014) | 0.49 | -0.022 (-0.053-0.009) | 0.16 |
| 3 | **0.092 (0.070-0.114)** | **<0.001** | **0.104 (0.080-0.128)** | **<0.001** | **0.033 (0.014-0.052)** | **0.001** | 0.020 (-0.003-0.043) | 0.08 | **0.088 (0.067-0.109)** | **<0.001** | **0.095 (0.063-0.127)** | **<0.001** |
| 4 | **0.037 (0.005-0.070)** | **0.02** | 0.028 (-0.011-0.068) | 0.16 | 0.019 (-0.006-0.044) | 0.14 | 0.005 (-0.023-0.033) | 0.73 | **0.087 (0.069-0.106)** | **<0.001** | **0.095 (0.065-0.124)** | **<0.001** |
| 5 | -0.004 (-0.035-0.027) | 0.79 | 0.021 (-0.028-0.071) | 0.39 | **-0.048 (-0.079, -0.016)** | **0.003** | -0.058 (-0.109- -0.008) | 0.02 | **0.124 (0.104-0.145)** | **<0.001** | **0.131 (0.095-0.166)** | **<0.001** |
| 6 | 0.037 (-0.003-0.077) | 0.07 | 0.010 (-0.034-0.055) | 0.66 | -0.004 (-0.027-0.018) | 0.70 | -0.007 (-0.057-0.043) | 0.78 | **0.095 (0.078-0.112)** | **<0.001** | **0.103 (0.074-0.133)** | **<0.001** |
| **Proportion of population in cluster** |  |  |  |  |  |  |  |  |  |  |  |  |
| Adult (over 15 years), per tertile | 0.008 (-0.006-0.022) | 0.27 | 0.004 (-0.016-0.025) | 0.67 | -0.006 (-0.014-0.001) | 0.11 | -0.008 (-0.025-0.009) | 0.37 | -0.003 (-0.015-0.010) | 0.67 | -0.003 (-0.020-0.015) | 0.76 |
| Male | 0.005 (-0.007-0.016) | 0.43 | -0.001 (-0.011-0.010) | 0.91 | 0.004 (-0.002-0.009) | 0.18 | -0.002 (-0.012-0.008) | 0.66 | -0.006 (-0.013-0.001) | 0.09 | -0.007 (-0.015-0.002) | 0.12 |
| Migratory, per quintile | 0.002 (-0.007-0.012) | 0.63 | -0.002 (-0.017-0.012) | 0.77 | -0.003 (-0.008-0.001) | 0.14 | 0.001 (-0.009-0.011) | 0.90 | -0.001 (-0.007-0.006) | 0.78 | 0.003 (-0.011-0.016) | 0.71 |
| Speaking minority languages‡ | **-0.038 (-0.051, -0.024)** | **<0.001** | **-0.023 (-0.041, -0.005)** | **0.01** | 0.004 (-0.007-0.015) | 0.46 | 0.012 (0.001-0.023) | 0.04 | -0.011 (-0.027-0.005) | 0.18 | -0.010 (-0.027-0.007) | 0.24 |
| Minority religion§ | **0.035 (0.019-0.050)** | **<0.001** | 0.012 (-0.016-0.040) | 0.40 | 0.001 (-0.010--0.013) | 0.80 | -0.003 (-0.014-0.009) | 0.66 | -0.005 (-0.025-0.014) | 0.59 | 0.003 (-0.015-0.021) | 0.72 |
| Polygamous¶ | 0.021 (-0.002-0.044) | 0.07 | 0.002 (-0.025-0.029) | 0.89 | NA |  | NA |  | -0.007 (-0.029-0.016) | 0.56 | -0.007 (-0.021-0.008) | 0.37 |
| **Population density**** | -0.0002 (-0.001-0.001) | 0.62 | 0.00002 (-0.001-0.001) | 0.96 | -0.0001 (-0.001-0.0003) | 0.63 | -0.0002 (-0.001-0.0003) | 0.46 | -0.001 (-0.003-0.001) | 0.47 | -0.001 (-0.003-0.001) | 0.59 |
| **Number CDDs trained**†† | -0.001 (-0.012-0.010) | 0.91 | 0.007 (-0.010-0.024) | 0.41 | -0.001 (-0.003-0.001) | 0.37 | -0.001 (-0.004-0.002) | 0.47 | NA |  | NA |  |
| **Sensitization activities** |  |  |  |  |  |  |  |  |  |  |  |  |
| Community meetings | -0.006 (-0.018-0.006) | 0.36 | -0.017 (-0.034-0.001) | 0.06 | **0.001 (0.001-0.002)** | **<0.001** | 0.001 (-0.001-0.003) | 0.20 | 0.002 (-0.003-0.007) | 0.35 | 0.004 (-0.002-0.009) | 0.20 |
| Public dialogue event | **0.005 (0.002-0.007)** | **0.001** | 0.003 (-0.001-0.006) | 0.17 | dropped |  | dropped |  | 0.0002 (-0.004-0.004) | 0.93 | -0.0004 (-0.004-0.003) | 0.81 |
| Printed IEC materials, quartiles | -0.002 (-0.015-0.011) | 0.79 | -0.019 (-0.043-0.006) | 0.14 | 0.0001 (-0.004-0.004) | 0.95 | -0.002 (-0.007-0.003) | 0.38 | -0.0002 (-0.014-0.014) | 0.98 | 0.005 (-0.013-0.024) | 0.59 |
| Door to door visits, quartiles | -0.006 (-0.016-0.003) | 0.20 | -0.003 (-0.012-0.005) | 0.48 | 0.005 (-0.0003-0.011) | 0.06 | -0.001 (-0.009-0.008) | 0.90 | 0.004 (-0.003-0.011) | 0.27 | 0.006 (-0.002-0.013) | 0.14 |
| Radio, quartiles | **0.039 (0.012-0.065)** | **0.005** | **0.033 (0.007-0.058)** | **0.01** | no activity |  | no activity |  | no activity |  | no activity |  |
| Other mass media | no activity |  | no activity |  | dropped |  | dropped |  | no activity |  | no activity |  |

* Number of observations per site reflect 20 intervention clusters per site multiplied by six MDA rounds. Four clusters in MDA round 5 were omitted from the India data due to COVID-19 related government shutdown orders.

† Difference in proportion treated per one unit increase^.^

‡ Three categories in Benin and Malawi, four in India. Majority language: Pedah, Sahoue, Watchi, Mina, Adja, or Xwla in Benin; Chiyao in Malawi; Tamil in India. Minority language: Fon or other in Benin; Chichewa or other in Malawi; Telegu, Urdu, Hindi or other in India.

§ Majority religion: Christianity in Benin; Islam in Malawi; Hinduism in India. Minority religion: Islam, Voodoo, or traditional religion in Benin; Christianity or other in Malawi; Christianity, Islam, or other in India.

¶ Not assessed in India. Variable is grouped as “greater than median” or “less than or equal to median”.

** Population density per km^2^ per 100 people.

†† Data not available for all MDA rounds in Malawi.

**S5. Table 2.** **Individual-level correlates of non-treatment amongst adults in DeWorm3 intervention clusters**

|  | ***Benin*** | | | | ***India*** | | | | ***Malawi*** | | | |
| --- | --- | --- | --- | --- | --- | --- | --- | --- | --- | --- | --- | --- |
|  | ***Unadjusted (N = 168,615)*** | | ***Adjusted (N = 165,058)*** | | ***Unadjusted (N = 298,805)*** | | ***Adjusted (N = 298,237)*** | | ***Unadjusted (N = 154,682)*** | | ***Adjusted (N = 153,970)*** | |
| ***Predictors*** | ***OR (95% CI)*** | ***P-value*** | ***aOR (95% CI)*** | ***P-value*** | ***OR (95% CI)*** | ***P-value*** | ***aOR (95% CI)*** | ***P-value*** | ***OR (95% CI)*** | ***P-value*** | ***aOR (95% CI)*** | ***P-value*** |
| **Individual factors** |  |  |  |  |  |  |  |  |  |  |  |  |
| **Age (per five years)** | 0.89 (0.88–0.90) | **<0.001** | 0.92 (0.91–0.93) | **<0.001** | 0.84 (0.84–0.84) | **<0.001** | 0.94 (0.93–0.95) | **<0.001** | 0.80 (0.80–0.81) | **<0.001** | 0.84 (0.83–0.85) | **<0.001** |
| **Sex** |  |  |  |  |  |  |  |  |  |  |  |  |
| Female | 0.81 (0.77–0.85) | **<0.001** | 0.84 (0.80–0.88) | **<0.001** | 0.94 (0.89–1.00) | 0.063 | 1.02 (0.97–1.07) | 0.431 | 0.15 (0.14–0.16) | **<0.001** | 0.23 (0.22–0.24) | **<0.001** |
| Male | 1.0 |  | 1.0 |  | 1.0 |  | 1.0 |  | 1.0 |  | 1.0 |  |
| **Migratory status** |  |  |  |  |  |  |  |  |  |  |  |  |
| Migratory* | 5.06 (4.74–5.40) | **<0.001** | 4.31 (4.03–4.61) | **<0.001** | 7.50 (7.03–8.00) | **<0.001** | 5.34 (5.01–5.69) | **<0.001** | 9.11 (8.49–9.78) | **<0.001** | 6.20 (5.80–6.64) | **<0.001** |
| Non-migratory | 1.0 |  | 1.0 |  | 1.0 |  | 1.0 |  | 1.0 |  | 1.0 |  |
| **Marital status** |  |  |  |  |  |  |  |  |  |  |  |  |
| Unmarried | 1.0 |  | 1.0 |  | 1.0 |  | 1.0 |  | 1.0 |  | 1.0 |  |
| Married | 0.68 (0.65–0.71) | **<0.001** | 0.87 (0.83–0.91) | **<0.001** | 0.43 (0.43–0.43) | **<0.001** | 0.56 (0.54–0.59) | **<0.001** | 1.03 (1.03–1.03) | **<0.001** | 0.91 (0.86–0.96) | **0.001** |
| Married (polygamous)† | 0.59 (0.50–0.69) | **<0.001** | 0.75 (0.64–0.87) | **<0.001** | - |  | - |  | 1.80 (1.80–1.80) | **<0.001** | 1.15 (1.04–1.26) | **0.005** |
| Marital status unknown | 8.43 (7.37–9.65) | **<0.001** | 2.19 (1.76–2.74) | **<0.001** | 16.26 (10.66–24.80) | **<0.001** | 3.78 (1.81–7.91) | **<0.001** | 30.10 (21.52–42.10) | **<0.001** | 2.72 (1.94–3.82) | **<0.001** |
| **Education level** |  |  |  |  |  |  |  |  |  |  |  |  |
| Less than primary school | 1.0 |  | 1.0 |  | 1.0 |  | 1.0 |  | 1.0 |  | 1.0 |  |
| Any primary or middle school education | 1.18 (1.11–1.25) | **<0.001** | 1.04 (0.98–1.10) | 0.165 | 0.98 (0.92–1.04) | 0.538 | 0.92 (0.87–0.98) | **0.013** | 1.89 (1.79–2.01) | **<0.001** | 1.11 (1.05–1.17) | **<0.001** |
| Any secondary or higher secondary education | 1.25 (1.19–1.32) | **<0.001** | 0.97 (0.92–1.03) | 0.329 | 1.82 (1.69–1.95) | **<0.001** | 1.32 (1.23–1.43) | **<0.001** | 3.71 (3.35–4.10) | **<0.001** | 1.34 (1.22–1.47) | **<0.001** |
| Any higher/tertiary education | 1.71 (1.58–1.85) | **<0.001** | 1.24 (1.14–1.35) | **<0.001** | 4.02 (3.71–4.36) | **<0.001** | 1.92 (1.75–2.11) | **<0.001** | 19.53 (11.52–33.10) | **<0.001** | 2.93 (1.82–4.72) | **<0.001** |
| Other education level | 1.75 (1.30–2.35) | **<0.001** | 1.47 (1.10–1.98) | **0.010** | 2.78 (1.54–5.05) | **0.001** | 2.12 (1.26–3.58) | **0.005** | 3.55 (2.50–5.04) | **<0.001** | 1.17 (0.85–1.61) | 0.345 |
| Education level unknown | 11.61 (10.09–13.35) | **<0.001** | 1.49 (1.18–1.89) | **0.001** | 41.59 (26.93–64.24) | **<0.001** | 3.60 (1.80–7.21) | **<0.001** | 10.38 (8.74–12.31) | **<0.001** | 2.57 (2.20–3.01) | **<0.001** |
| **Household factors** |  |  |  |  |  |  |  |  |  |  |  |  |
| **Wealth quintile** |  |  |  |  |  |  |  |  |  |  |  |  |
| Lowest quintile | 1.0 |  | 1.0 |  | 1.0 |  | 1.0 |  | 1.0 |  | 1.0 |  |
| Low quintile | 1.04 (0.96–1.13) | 0.339 | 1.07 (0.99–1.16) | 0.092 | 1.19 (1.07–1.33) | **0.001** | 0.82 (0.75–0.90) | **<0.001** | 1.09 (0.98–1.22) | 0.121 | 0.98 (0.89–1.07) | 0.599 |
| Medium quintile | 0.88 (0.82–0.95) | **0.002** | 0.90 (0.83–0.97) | **0.009** | 1.30 (1.17–1.44) | **<0.001** | 0.81 (0.75–0.89) | **<0.001** | 0.99 (0.89–1.11) | 0.856 | 0.91 (0.84–1.00) | **0.043** |
| High quintile | 0.87 (0.81–0.94) | **0.001** | 0.89 (0.82–0.96) | **0.003** | 1.29 (1.17–1.43) | **<0.001** | 0.77 (0.70–0.84) | **<0.001** | 1.01 (0.90–1.12) | 0.913 | 0.94 (0.86–1.02) | 0.134 |
| Highest quintile | 0.83 (0.77–0.90) | **<0.001** | 0.79 (0.73–0.86) | **<0.001** | 1.58 (1.43–1.75) | **<0.001** | 0.82 (0.75–0.90) | **<0.001** | 1.23 (1.10–1.37) | **<0.001** | 0.92 (0.84–1.01) | **0.068** |
| **Household language** |  |  |  |  |  |  |  |  |  |  |  |  |
| Minority language | 1.54 (1.43–1.67) | **<0.001** | 1.49 (1.38–1.61) | **<0.001** | 1.67 (1.42–1.97) | **<0.001** | 1.39 (1.21–1.59) | **<0.001** | 2.68 (2.32–3.10) | **<0.001** | 1.53 (1.30–1.79) | **<0.001** |
| Majority language‡ | 1.0 |  | 1.0 |  | 1.0 |  | 1.0 |  | 1.0 |  | 1.0 |  |
| **Household religion** |  |  |  |  |  |  |  |  |  |  |  |  |
| Minority religion | 0.98 (0.93–1.04) | 0.506 | 0.96 (0.91–1.01) | 0.127 | 1.58 (1.35–1.84) | **<0.001** | 1.30 (1.14–1.48) | **<0.001** | 1.76 (1.54–2.01) | **<0.001** | 0.96 (0.83–1.10) | 0.548 |
| Majority religion§ | 1.0 |  | 1.0 |  | 1.0 |  | 1.0 |  | 1.0 |  | 1.0 |  |
| **Household population density (1 000 population per half Km)** | 0.98 (0.96–1.00) | **0.014** | 0.98 (0.96–0.99) | **0.008** | 1.05 (1.03–1.07) | **<0.001** | 1.01 (1.00–1.03) | 0.110 | 1.06 (1.02–1.09) | **0.002** | 1.01 (0.98–1.03) | 0.658 |
| *aOR* Adjusted odds ratio, *OR* odds ratio, *CI* confidence interval. | | | | | | | | | | | | |
| * Defined as living in the household <6 months in the year in the previous year. | | | | | | | | | | | | |
| † Not assessed in India. | | | | | | | | | | | | |
| ‡ Majority language is defined as Pedah, Sahoue, Watchi, Mina, Adja, and Xwla in Benin, Tamil in India and Chiyao in Malawi. | | | | | | | | | | | | |
| § Majority religion is defined as Christianity in Benin, Hinduism in India and Islam in Malawi. | | | | | | | | | | | | |
